# Effects of Antimalarial Drugs on Neuroinflammation-Potential Use for Treatment of COVID-19-Related Neurologic Complications

**DOI:** 10.1007/s12035-020-02093-z

**Published:** 2020-09-08

**Authors:** Wei-Yi Ong, Mei-Lin Go, De-Yun Wang, Irwin Kee-Mun Cheah, Barry Halliwell

**Affiliations:** 1 Department of Anatomy, Yong Loo Lin School of Medicine, National University of Singapore, Singapore, 119260 Singapore; 2 Neurobiology Programme, Life Sciences Institute, National University of Singapore, Singapore, 119260 Singapore; 3 Department of Pharmacy, Faculty of Science, National University of Singapore, Singapore, 119260 Singapore; 4 Department of Otolaryngology, Yong Loo Lin School of Medicine, National University of Singapore, Singapore, 119260 Singapore; 5 Department of Biochemistry, Yong Loo Lin School of Medicine, National University of Singapore, Singapore, 119260 Singapore

**Keywords:** SARS-CoV-2, COVID-19, COVID, SARS, Coronavirus, Antimalarials, Statins, Chloroquine, Hydroxychloroquine, Quinacrine, Aminoacridine, Cytosolic phospholipase A_2_, cPLA_2_, Secretory phospholipase A_2_, sPLA_2_ IID, TNF-α, DMT1, Iron transport, Arachidonic acid, Eicosanoids, Inflammation, Neuroinflammation, Microglia, Lysosomes, Oxidative stress, Free radical damage, Carotid body, Aortic body, Nucleus of the tractus solitarius, Glossopharyngeal nerve, Vagus nerve, Respiratory centre, Hippocampus, Stroke, Microvessels, Microcirculation, Brain endothelial cells, Vascular dementia

## Abstract

The SARS-CoV-2 virus that is the cause of coronavirus disease 2019 (COVID-19) affects not only peripheral organs such as the lungs and blood vessels, but also the central nervous system (CNS)—as seen by effects on smell, taste, seizures, stroke, neuropathological findings and possibly, loss of control of respiration resulting in silent hypoxemia. COVID-19 induces an inflammatory response and, in severe cases, a cytokine storm that can damage the CNS. Antimalarials have unique properties that distinguish them from other anti-inflammatory drugs. (A) They are very lipophilic, which enhances their ability to cross the blood-brain barrier (BBB). Hence, they have the potential to act not only in the periphery but also in the CNS, and could be a useful addition to our limited armamentarium against the SARS-CoV-2 virus. (B) They are non-selective inhibitors of phospholipase A_2_ isoforms, including cytosolic phospholipase A_2_ (cPLA_2_). The latter is not only activated by cytokines but itself generates arachidonic acid, which is metabolized by cyclooxygenase (COX) to pro-inflammatory eicosanoids. Free radicals are produced in this process, which can lead to oxidative damage to the CNS. There are at least 4 ways that antimalarials could be useful in combating COVID-19. (1) They inhibit PLA_2._ (2) They are basic molecules capable of affecting the pH of lysosomes and inhibiting the activity of lysosomal enzymes. (3) They may affect the expression and Fe^2+^/H^+^ symporter activity of iron transporters such as divalent metal transporter 1 (DMT1), hence reducing iron accumulation in tissues and iron-catalysed free radical formation. (4) They could affect viral replication. The latter may be related to their effect on inhibition of PLA_2_ isoforms. Inhibition of cPLA_2_ impairs an early step of coronavirus replication in cell culture. In addition, a secretory PLA_2_ (sPLA_2_) isoform, PLA2G2D, has been shown to be essential for the lethality of SARS-CoV in mice. It is important to take note of what ongoing clinical trials on chloroquine and hydroxychloroquine can eventually tell us about the use of antimalarials and other anti-inflammatory agents, not only for the treatment of COVID-19, but also for neurovascular disorders such as stroke and vascular dementia.

## COVID-19 Outbreak and the Use of Chloroquine and Hydroxychloroquine Against COVID-19

The first cases of coronavirus disease 2019 (COVID-19) were reported near the end of 2019 in Wuhan, China. In March 2020, BBC and CNN news reported President Donald Trump’s ‘fantastic’ treatment for coronavirus, chloroquine and hydroxychloroquine. As a result, hydroxychloroquine was snapped up by medical institutions at more than twice the typical pace, as US hospitals sought to build large inventories in anticipation of the medication’s potential use in patients with COVID-19 (https://www.bloomberg.com/news/articles/2020-03-20/hospitals-stockpile-malaria-drug-trump-says-could-treat-covid-19). Nevertheless, the US Food and Drug Administration cautions against the use of chloroquine and hydroxychloroquine for COVID-19 outside of a hospital setting or a clinical trial due to risk of heart rhythm problems. While there are some reports suggesting that hydroxychloroquine could be a promising treatment intervention there have been, as yet, no large-scale randomized controlled trials to support those claims. Chloroquine and hydroxychloroquine have been used for the treatment of malaria, lupus and rheumatoid arthritis for many years. From the literature, serious side-effects seem quite rare when they are used to treat these diseases, apart from hemolysis in some patients with glucose-6-phosphate dehydrogenase deficiency and possible ocular side effects [1]. In this paper, we point out why they and other antimalarials might be effective in combatting certain aspects of COVID-19 infection, and the clinician has to balance this with the risk of side effects.

## COVID-19 Is a Proinflammatory Condition

**Fig. 1:**
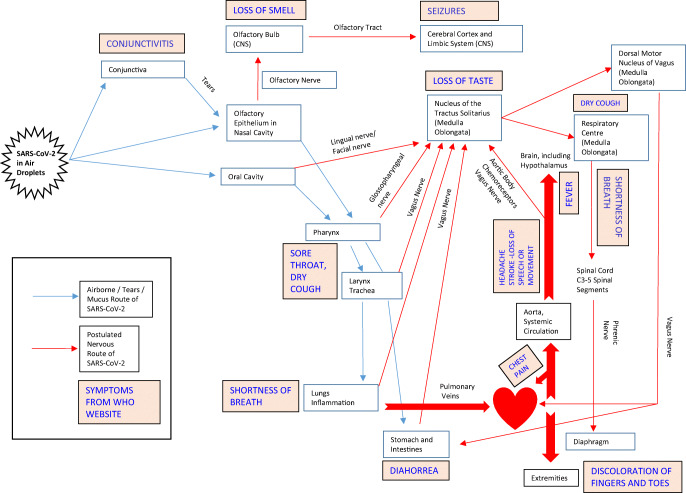
Airborne/tears/mucus route of spread of SARS-CoV-2, postulated nervous route of the virus and COVID-19 symptoms

The SARS-CoV-2 virus, which is the cause of COVID-19, is known to infect both the upper airways and the lungs. The virus replicates in the lungs and can spread to other parts of the body through the vascular and possibly lymphatic systems (Fig. 1). COVID-19 causes an imbalance between adaptive and innate immunity. On one hand, there is decreased function of immune cells such as lymphocytes and T cell exhaustion, [2] but on the other, there is increased response from macrophages that are a key part of the innate immune response [3]. In some cases, a dysfunctional and hyperactive immune response can trigger a cytokine storm that mediates widespread lung inflammation. It has been observed that patients with severe COVID-19 that require intensive care in hospitals have greater macrophage inflammatory protein 1 in the plasma [4] and monocyte-derived FCN1+ macrophage population in the bronchoalveolar fluid [5]. Not only can the virus spread through the bloodstream, it can also infect endothelial cells in the vessel walls [6]. This could lead to an inflammatory response in these cells and an increase in blood coagulation. Hence, COVID-19 may predispose patients to thrombotic disease in the venous and arterial circulations due to excessive inflammation, platelet activation, endothelial dysfunction and stasis [7].

The problems of COVID-19 are aggravated by pre-existing medical conditions, in particular metabolic and cardiovascular diseases [8]. A common feature of these diseases is high levels of cholesterol oxidation products or oxysterols in the bloodstream [9]. In particular, 7-ketocholesterol has marked ability to induce cytokine expression and inflammation in cells. It induces the expression of the cytokines VEGF, IL-6 and IL-8 through the AKT-PKCζ-NFκB, p38 MAPK and ERK pathways in ARPE-19 cells [10] and increases IL-1β, TNF-α, IL-6 and IL-1β secretion and inflammation in these cells [11]. The oxysterol also markedly increases the formation and activation of NLRP3 inflammasomes and elevates IL-1β levels in mouse carotid arterial endothelial cells [12]. This could aggravate damage to endothelial cells that are exposed to SARS-CoV-2. Serum 7-ketocholesterol level is associated with increased risk of cardiovascular events and mortality in patients with stable coronary artery disease [13]. Likewise, elevated plasma 7-ketocholesterol level is associated with the incidence of cardiovascular disease in a population-based cohort [14]. Increased 7-ketocholesterol levels are found in patients with diabetes or hypercholesterolemia [15].

Another factor that could be related to neuroinflammation is dysregulation of iron homeostasis [16]. Many patients with COVID-19 have increased iron, as reflected by an increased level of the iron storage protein, ferritin [17]. Excess iron is stored in cells by being bound to ferritin, but the latter can be downregulated by high levels of oxidative stress, resulting in release of iron ions that are capable of catalysing free radical reactions [18]. Iron transport across enterocytes in the intestines and across the blood-brain barrier (BBB) is dependent on the iron transporter, DMT1 [19]. The gene encoding the latter contains putative AP-1 and NF-κB binding sites, a possible interferon γ-responsive element and a hypoxia-inducible factor-1 (HIF-1)-like motif [20, 21]. It is therefore possible that increased AP-1 or NF-κB activation during a COVID-19-induced cytokine storm could result in activation of these sites on the DMT1 promoter, resulting in increased expression of the iron transporter and iron accumulation in tissues.

The ability of COVID-19 to produce a cytokine storm and inflammation might be at the centre of the pathogenicity of the virus, yet little is known about the component(s) of SARS-CoV-2 that is pro-inflammatory. In the case of HIV, it is the gp120 protein that has been found to be pro-inflammatory in neurons [22]. Research is urgently needed to identify the pro-inflammatory component(s) of SARS-CoV-2.

## COVID-19 Affects the CNS

Besides peripheral effects, COVID-19 has also been reported to cause neurologic dysfunction (Fig. 1). Anosmia, stroke, paralysis, cranial nerve deficits, encephalopathy, delirium meningitis and seizures are some of the complications in patients with COVID-19 [23–25]. Many patients who have contracted COVID-19 report a loss in smell or taste [26]. The sense of smell originates from olfactory neurons in the olfactory epithelium in the upper part of the nasal cavity. It travels via bundles of the olfactory nerves to the olfactory bulb and thereafter along the olfactory tract to the septum, cerebral cortex and other parts of the brain. Damage to either the olfactory neurons or their supporting cells and/or the olfactory bulb could lead to anosmia [27]. Recent MRI studies have shown transient changes in the olfactory bulb that accompany COVID-19-related anosmia, consistent with CNS involvement in some of the patients [28, 29]. Study of the intracerebral distribution of gold nanoparticles after inhalation exposure in rats shows that the nanoparticles are found in nuclei connected to the olfactory and limbic systems, including the olfactory bulb, hippocampus, frontal cortex, striatum, entorhinal cortex and septum [30, 31]. This could be an indication of the potential areas of spread of viruses from the olfactory bulb. The SARS-CoV virus, which is taxonomically very similar to SARS-CoV-2, is able to enter the brain via the olfactory bulb, and infection results in rapid, transneuronal spread to connected areas of the brain. CNS infection is thought to be the main cause of death in mice experimentally infected with the SARS-CoV virus since intracranial inoculation with low doses of this virus results in lethality, even though little infection is detected in the lungs [32]. Besides the olfactory axonal route, SARS-CoV-2 may pass from non-neuronal olfactory epithelium cells directly to the cerebrospinal fluid surrounding olfactory nerve bundles and enter the brain [33]. Other viruses, e.g. herpesvirus-6 [34] or rabies virus, are also known to hijack existing vesicular axonal transport machineries to travel within the brain (reviewed in [35]).

Taste is also often affected by COVID-19. This modality is conveyed via axons in the facial, glossopharyngeal and vagus nerves that terminate in the nucleus of the tractus solitarius in the medulla oblongata. The loss of taste strongly suggests damage to this nucleus, possibly via retrograde transport of the virus along axons. It is interesting to note that the caudal part of the nucleus of the tractus solitarius receives visceral afferent signals from the airway that mediate the cough reflex, and it is possible that irritation of this nucleus could contribute to coughing, which is a common symptom of patients with COVID-19. Chemoreceptors in the carotid body or aortic body in the walls of the internal carotid artery or the aorta sense the level of oxygen or carbon dioxide in the blood and convey these signals via the glossopharyngeal and vagus nerves to the nucleus of the tractus solitarius. Moreover, afferent fibres in the vagus nerve convey the sense of stretch from the lungs to the same nucleus, and these are involved in the reflex control of respiratory rate. It is possible that interference with chemoreceptors as a result of vasculitis or inflammatory changes in the vessel walls [6, 36] and/or the nucleus of the tractus solitarius [37] could lead to loss of feedback control to regulate the oxygenation in the blood and might explain the ‘silent hypoxia’ or ‘happy hypoxia’ even without lung exudates in many patients with COVID-19 [33, 38]. Localized perivascular and interstitial encephalitis with neuronal cell loss and axon degeneration in the dorsal motor nucleus of the vagus nerve, trigeminal nucleus and nucleus tractus solitarii have been detected in the brains of patients with COVID-19 [37]. COVID-19 may also be associated with unexplained altered mental status or loss of consciousness in 13% of patients [39]. Study of 26 critically ill hospitalized SARS-CoV-2 patients with unexplained neurological symptoms found that 5 patients had EEGs with biphasic delta periodic discharges indicative of CNS injury [40].

Previous studies have shown the ability of SARS-CoV to induce neuronal death in mice by invading the brain via the olfactory epithelium [32]. Entry of coronavirus into the primate CNS has also been demonstrated after peripheral infection [41]. SARS-CoV is present in brain tissue from human autopsies, and tissue oedema and neuronal degeneration were prominent findings in sections [42]. Moreover, the SARS genomic sequence has been detected by in situ hybridization histochemistry in brain neurons of a patient infected by SARS-CoV [42]. In the current pandemic, gene sequencing confirmed the presence of SARS-CoV-2 in the cerebrospinal fluid of a 56-year-old patient with novel coronavirus in Beijing Ditan Hospital [43]. COVID-19 effects on the brain may be an extension of its effects on the periphery. The same cytokines that cause inflammation in the periphery, most commonly, IL-1β, TNF-α and IL-6, may also induce neuroinflammation in the brain [44]. Brain inflammation has been shown to underlie, at least in part, the CNS damage associated with infection by West Nile, Zika and herpes simplex viruses, conditions in which long-lasting inflammatory processes develop within the CNS [35]. Intracranial cytokine storm has been implicated in causing COVID-19-related acute necrotizing haemorrhagic encephalopathy. This can lead to symmetric thalamic encephalitis as well as similar symmetric lesions in other brain regions [45]. Cerebral microbleeds and leukoencephalopathy have also been detected in critically ill patients with COVID-19 [46]. It is likely that the paucity of brain imaging studies being performed in critically ill COVID-19 patients may be an important factor in the lack of wider recognition of such complications.

Besides a possible route to the brain via peripheral nerves, the SARS-CoV-2 virus might gain entry into the brain by infecting endothelial cells [6]. Observations of viral-like particles in brain capillary endothelium and active budding across endothelial cells suggest a role of brain microvessels as a route of SARS-CoV-2 entry into the brain [47]. Damage to cerebral blood vessels may also predispose the patients to stroke [48] and possibly vascular dementia. The intense systemic inflammatory response linked to viral infections may lead to damage to the blood-brain barrier (BBB), thus allowing more virus or peripheral cytokines including TNF-α (which is neurotoxic [49]) to enter the brain where they may trigger or exacerbate neuroinflammation. Since the hippocampus is well known to be particularly susceptible to global ischemia, this structure could also be damaged by prolonged silent hypoxemia in patients with severe COVID-19, with resultant effects on declarative memory.

Like the SARS-CoV virus, SARS-CoV-2 uses a spike protein S1 to enable the virus to gain entry to cells by attaching to the host ACE2 receptor [50] and TMPRSS2 priming on the cell membrane. ACE2 receptor is highly expressed in the lungs, heart, liver and CNS, which includes the olfactory bulb while TMPRSS2 is expressed in the liver, peripheral nervous system and CNS [51].

## Phospholipase A_2_ (PLA_2_) Plays a Critical Role in Coronavirus Replication and Pathogenicity

The enzyme cytosolic phospholipase A_2_ (cPLA_2_) is a key enzyme in innate immunity. It acts on membrane glycerophospholipids to release a free fatty acid (arachidonic acid) and a lysophospholipid. Arachidonic acid can be further metabolized by cyclooxygenases (COX) to prostaglandins and other pro-inflammatory mediators. Hence, inhibition of cPLA_2_ has been found by many groups to have an anti-inflammatory and neuroprotective effect (for reviews, see [52, 53]). Increased cPLA_2_ activity is found in microglial cells that are stimulated by lipopolysaccharide (LPS) or interferon gamma (IFNγ). On the other hand, LPS- and IFNγ-induced production of reactive oxygen species (ROS) and nitric oxide (NO) are inhibited by a selective inhibitor of cPLA_2_, arachidonyl trifluoromethyl ketone (AACOCF_3_) [54]. ROS such as superoxide radicals, hydrogen peroxide and hydroxyl radicals and reactive nitrogen species such as nitric oxide or peroxynitrite can cause damage to cellular components if present in excess [18]. cPLA_2_ could be a link between the increased cytokine production that is found in COVID-19 and inflammation and oxidative stress. MAPKs and NF-κB have been shown to be involved in IL-1β-induced cPLA_2_ expression in canine tracheal smooth muscle cells [55], and TNFα has been shown to induce cPLA_2_ expression in lung epithelial cells [56]. A pathway for cPLA_2_ activation via IL-13 has also been described [57]. Since cPLA_2_ expression is increased by cytokines and itself forms eicosanoids that are pro-inflammatory, it could be a key enzyme in a feed-forward cycle to propagate a cytokine storm.

As a cellular defence against such oxidative damage, arachidonic acid that is formed by cPLA_2_ is bound by the lipocalin apolipoprotein D (apoD) and prevented from forming toxic lipid peroxides [58]. It is interesting to note that overexpression of human apoD in neurons of Thy-1/apoD transgenic mice resulted in a threefold increase in the number of mice surviving coronavirus (HCoV-OC43) infection [59]. cPLA_2_ is also found to be essential for coronavirus replication. Inhibition of this enzyme is reported to impair an early step of coronavirus replication in cell culture, probably due to interference with the formation of lysophospholipids [60].

A critical role of another member of the PLA_2_ superfamily, secretory phospholipase A_2_ (sPLA_2_) Group IID in age-related susceptibility to SARS-CoV infection, has also been reported. Strikingly, infection of mice lacking PLA2G2D (Pla2g2d (^−^/^−^) mice) converted a uniformly lethal infection to a nonlethal one (> 80% survival), together with *enhanced* antivirus T cell responses and diminished lung damage [61].

## Effects of Antimalarials on Phospholipase A_2_

**Fig. 2:**
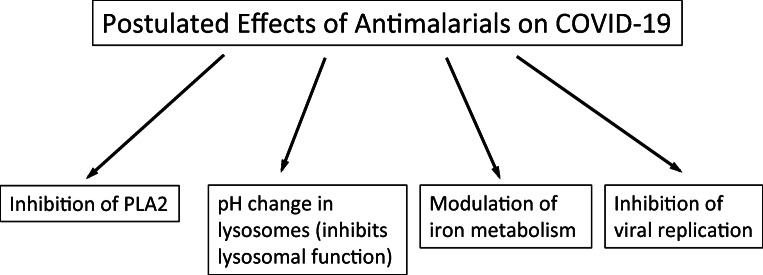
Potential effects of antimalarials on COVID-19

**Fig. 3:**
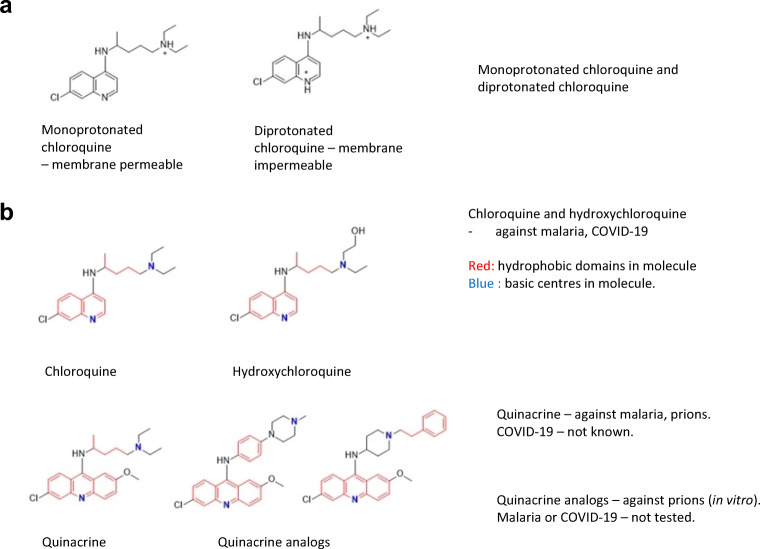
Structure of chloroquine, hydroxychloroquine, quinacrine and quinacrine derivatives. **a** Monoprotonated chloroquine and diprotonated chloroquine. **b** Red—hydrophobic domains in molecules; blue—basic centres in molecules

**Fig. 4:**
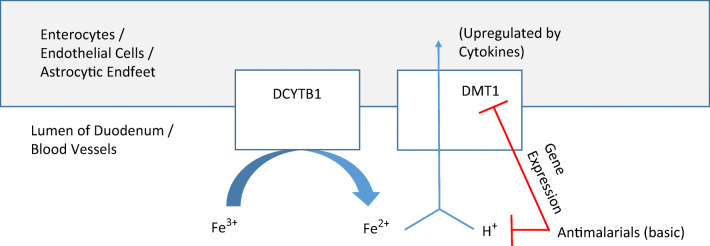
Effect of quinacrine on iron transport through DMT1 in the duodenum and blood-brain interface. Ferric iron is converted to ferrous iron by duodenal cytochrome B (DCTB). Thereafter ferrous iron is taken up into enterocytes, endothelial cells or astrocytes by divalent metal transporter-1 (DMT1) together with a proton (H^+^). Antimalarials reduce the number of protons available for the H^+^/Fe^2+^ symporter activity of DMT1. Quinacrine also reduces DMT1 expression itself

How might antimalarials help in COVID-19? There are at least four possibilities: (1) They inhibit PLA_2_; (2) they are basic molecules capable of affecting the pH of lysosomes and inhibiting the activity of lysosomal enzymes; (3) they may affect the expression and Fe^2+^/H^+^ symporter activity of iron transporters such as divalent metal transporter 1 (DMT1), hence reducing iron accumulation in tissues and iron-catalysed free radical formation [62]; and (4) they may affect viral replication (Figs. 2, 3 and 4). Chloroquine, hydroxychloroquine and quinacrine are known to be non-selective inhibitors of different PLA_2_ isoforms. They enter and accumulate in lysosomes and are thought to act by altering the pH of lysosomes. This results in inhibition of lysosomal enzymes in the malarial parasite and interference with parasite feeding [63]. Among the lysosomal enzymes are a wide range of proteases, lipases and nucleases. An inhibitory effect on malarial PLA_2_ has been shown with chloroquine, quinine and arteether (derived from artemisinin) [64]. Moreover, chloroquine and mepacrine (quinacrine) inhibit cPLA_2_ in rat heart homogenates [65]. Antimalarial drugs inhibit PLA_2_ activation and induction of IL-1β and TNFα in macrophages. Chloroquine, quinacrine and, to a lesser extent, hydroxychloroquine inhibit arachidonic acid release and eicosanoid formation induced by phorbol diester in macrophages. This effect is due to inhibition of arachidonic-acid preferring PLA_2_ (cPLA_2_) [66]. Besides inhibition of PLA_2_ activity, Northern blot analyses showed that quinacrine reduced cPLA_2_ mRNA expression in the rat hippocampus after kainate-induced excitotoxic injury [67]. It also reduced cPLA_2_ immunoreactivity and protected neurons from cellular injury in hippocampal slice cultures after kainate treatment [68]. Moreover, quinacrine reduced cPLA_2_ immunoreactivity in glial cells and protected neurons from damage after intracerebroventricular lipopolysaccharide injection [69]. Quinacrine has been found to block the binding of the transcription factor AP-1 to its binding site on DNA and could reduce AP-1-induced gene expression in target cells [70], i.e. it both inhibits and deceases the expression of cPLA_2_. All isoforms of bovine brain PLA_2_ are strongly inhibited by antimalarial drugs in a dose-dependent manner. Chloroquine, quinacrine, hydroxychloroquine and quinine inhibit bovine brain cPLA_2_ with IC_50_ values of 125, 200, 185 and 250 μM [53, 71]. Chloroquine or quinacrine have been found to reduce cPLA_2_ immunoreactivity in hippocampal slice cultures following kainate treatment. Interestingly, the effects on cPLA_2_ expression are observed at 10 μM chloroquine or quinacrine, i.e. concentrations that are about an order of magnitude lower than the IC_50_ of these inhibitors for cPLA_2_. These drugs may therefore not only inhibit cPLA_2_ enzymatic activity at higher concentration but also induce downregulation of cPLA_2_ protein expression at lower concentrations [71]. Other isoforms of PLA_2_, such as sPLA_2_, are also important in inflammation, but even then, their ability to induce arachidonic acid release is dependent on cPLA_2_ [72]. Gene knockout of cPLA_2_ results in downregulation of its downstream enzyme cyclooxygenase-2 (COX-2) leading to a reduction in eicosanoid production including the pro-inflammatory PGE2. sPLA_2_ and calcium-independent phospholipase A2 (iPLA2) do not compensate for the loss of brain cPLA2 [73].

The CNS penetration ability of drugs has been reported to be a critical factor in the treatment of SARS-CoV-2 brain infection [74]. Among antimalarials, there is compelling evidence to support the BBB permeability of mefloquine, a quinolinemethanol that is structurally related to quinine [75, 76]. Less is known of the potential of chloroquine and hydroxychloroquine to transverse the BBB. Studies have shown that certain structural properties may be used to predict the BBB permeability of solutes [77]. One such set of empirical rules was articulated by Clark [78] and Lobell et al. [79] who stated that access into the central nervous system required molecules to fulfil the following threshold values: (i) molecular weight < 450; (ii) polar surface area (PSA) < 60–70 Å^2^; (iii) total number of N and O atoms < 6; (iv) distribution coefficient Log D 7.4 between 1 and 3; (v) clogP – (N + 0) > 0. Functional groups with N and O atoms and the H atoms attached to them contribute to the polar surface area of the molecule, and together, both parameters are surrogate measures of the hydrogen bonding capacity of the molecule. Lipophilicity is assessed from clogP, which is derived from the neutral (non-ionized) state of the molecule, and logD, which measures the contribution of both non-ionized and ionized states of the molecule at a stated pH, usually pH 7.4. Here, we obtained the parameters (i–v) of chloroquine and hydroxychloroquine and compared them with those of mefloquine (Table 1). The parameters of quinacrine were also included as this compound was found to possess a PAMPA Pe value that was indicative of BBB permeability [80]. As seen from Table 1, both chloroquine and hydroxychloroquine fulfilled the requirements stated for molecular weight, polar surface area and hydrogen bonding propensity (PSA, number of N, O atoms; clogP – [N + O]) required for CNS penetration. The size, lipophilicity and H bonding capability of hydroxychloroquine are, in fact, closely aligned to that of mefloquine, and the parameters obtained for quinacrine lend support to its ‘CNS +’ status as implicated from its PAMPA Pe.

**Table 1:** Assessment of the BBB permeability of mefloquine, chloroquine, hydroxychloroquine and quinacrine based on predictive physicochemical properties [78, 79]

Antimalarial	Molecular weight	PSA (Å^2^)^a^	Log D_7.4_^b^	Number of N and O atoms	cLogP^a^	clogP minus (N+O) > 0
Mefloquine	377	46.7	0.5	3	4.0	Yes
Chloroquine^c^	319	27.6	1.6	3	5.1	Yes
Hydroxychloroquine^c^	335	47.9	2.0	4	4.1	Yes
Quinacrine	399	36.9	2.3	4	6.2	Yes

^a^Determined from ChemDraw Professional, Ver 15

^b^Determined from ACD /Labs Version 12

^c^pKa values of chloroquine are 10.5 and 6.3. pKa values of hydroxychloroquine are 8.9 and 6.3. Determined from ACD/Labs Version 12

The ability of antimalarials to cross the BBB could be particularly important, in view of the neuroinvasive potential of SARS-CoV and SARS-CoV-2 and the observations noted above that many COVID-19 patients have loss of control of respiration and silent hypoxemia even without lung exudates [38, 81]. Rats that received a fimbria-fornix lesion showed increased cPLA_2_ in the septum (cell body of some of the transected axons), accompanied by an increase in the oxidative stress marker, 4-hydroxynonenal (4-HNE) [18]. The latter is a breakdown product of peroxidized arachidonic acid that has been released from membrane glycerophospholipids by the action of cPLA_2_. It contains reactive alkene and aldehyde groups and can form adducts with and inhibit the function of cellular proteins. Intraperitoneal injection of quinacrine to rats results in significant reduction of both cPLA_2_- and HNE-positive cells in the septum [82]. Observation of the brains of rats that have received prior intraperitoneal quinacrine injections showed that the brains were stained yellow due to the antimalarial drug, demonstrating clearly that it can cross the BBB. This confirms the findings of an early study in primates that quinacrine is able to enter almost all tissues in the body from the bloodstream and remain detectable for at least a week even after blood levels have subsided [83]. The ability of quinacrine and other antimalarials to function as cPLA_2_ inhibitors that are able to cross the BBB could enable them to act centrally within the brain, in addition to their peripheral effect, and could be a unique action among existing anti-inflammatory agents, which is perhaps shared with some of the brain-permeable statins [84–86].

## Effect of Antimalarials on pH and Lysosomal Enzymes

Chloroquine and hydroxychloroquine are lipophilic molecules as seen from their estimated clogP values (Table 1; Fig. 3). They are also strong bases with two ionizable basic groups—a weakly basic quinoline ring N (pKa 6.3) and a strongly basic side chain tertiary amine (pKa 10.5, 8.9) (Table 1). Chloroquine is a known lysosomotropic agent. It accumulates within lysosomes, which are highly acidic organelles. Accumulation within lysosomes is driven by the physicochemical character of these molecules. The monoprotonated state, which is the main species at physiological pH, diffuses across the lysosomal membranes and, once within the highly acidic environment of the lysosomes, acquires protons to become the less permeable diprotonated form. The latter is consequently trapped within the lysosomes, thus inducing an increase in the pH of the compartment due to the acquisition of protons to form the diprotonated state. Hence, enzyme activity or any metabolic process within the lysosomes that requires a low pH is impaired. For the malarial parasite, this translates to an interference with parasite feeding. This is one way by which chloroquine and related compounds exert their antimalarial properties. We have earlier synthesized and reported two acridine derivatives that have greater activity than quinacrine against prions [87]. These compounds retain the motif that is associated with lysosomotropic activity (lipophilic and with two basic centres). The structures of chloroquine, hydroxychloroquine, quinacrine and derivatives of quinacrine are shown in Fig. 3.

## Effect of Antimalarials on Iron Metabolism

Interference with iron accumulation has been postulated as one of the mechanisms by which chloroquine and hydroxychloroquine are beneficial against COVID-19 [62]. This could occur via an inhibitory effect of antimalarials on iron uptake into tissues (Fig. 4). An increase in brain iron level is found by nuclear microscopy of the rat hippocampus undergoing neuroinflammation after excitotoxic injury induced by kainate [88]. The increased iron in the brain is accompanied by increased expression of both a ferrireductase DCYTB1 [89] and DMT1 [89, 90]. Intraperitoneal injections of quinacrine resulted in reduced DMT1 expression and decreased numbers of ferric or ferrous iron–containing glial cells in the degenerating hippocampus after kainate lesions [90].

Basic drugs such as antimalarials are also able to affect iron uptake by inhibiting iron release from its transport protein, transferrin [91]. Moreover, DMT1 is a H^+^/Fe^2+^ symporter that needs a proton electrochemical potential gradient to drive the transport of iron from endosomes into the cytoplasm [92], and antimalarials could alter the pH environment that is necessary for DMT1 transporter activity in endosomes.

## Effect of Antimalarials on Viral Replication

It has been shown in cell culture that antimalarials can affect coronavirus replication (reviewed in [93]). For example, chloroquine was found to have significant effects on viral cell entry and replication in vitro [94]. The exact mechanism is unknown, although one possibility is that it may be related to inhibition of cPLA_2_, which as mentioned, was found to be essential for an early step in coronavirus replication [60].

## Summary and Perspectives for Future Development

COVID-19 is a pro-inflammatory-driven condition with loss of smell and taste, suggesting that it may affect the olfactory and gustatory systems and the brain. These effects may persist even after the virus has been cleared from the body [43]. Antimalarials could have a beneficial effect on COVID-19 through (1) PLA_2_ inhibition, (2) pH change in lysosomes, (3) modulation of iron metabolism [62] and (4) possible direct antiviral activity. At the same time, antimalarials have the ability to cross the BBB and thus be beneficial in alleviating COVID-19-induced changes in the CNS. Chloroquine and hydroxychloroquine were found to be effective in controlling neurosarcoidosis in human patients. The drugs may be beneficial in inexorable disease progression, in patients where corticosteroids are clearly contraindicated [95]. On the other hand, anti-inflammatory treatment with hydroxychloroquine for 18 months does not slow the rate of decline in early stage or mild Alzheimer’s disease [96]. Further studies are needed to determine the structure–activity relationships of antimalarials to improve their efficacy for treatment of COVID-19. Such research may also be fruitful in combatting other forms of neuroinflammation associated with age-related neurological disorders.

Non-randomized or randomized controlled trials on patients with COVID-19 have identified benefits in clinical and virological outcomes with chloroquine or hydroxychloroquine treatment [97–99]. Other studies have reported no positive effects [100]. One meta-analysis of publicly available clinical reports suggests that chloroquine derivatives are effective in reducing mortality by a factor of 3 in patients infected with COVID-19 [101]. Few studies, however, have looked at CNS effects. At the time of writing of this paper, there are 35 ongoing trials investigating the use of the antimalarial drugs such as chloroquine and hydroxychloroquine against COVID-19, with another 34 more registered [102]. It is important to take note of what these ongoing clinical trials can eventually tell us about the use of antimalarials and other anti-inflammatory agents [99], for the treatment not only of COVID-19 but also of neuroinflammation and neurological/neurovascular disorders such as stroke and vascular dementia.
